# Estimating Influenza Hospitalizations among Children

**DOI:** 10.3201/eid1201.050308

**Published:** 2006-01

**Authors:** Carlos G. Grijalva, Allen S. Craig, William D. Dupont, Carolyn B. Bridges, Stephanie J. Schrag, Marika K. Iwane, William Schaffner, Kathryn M. Edwards, Marie R. Griffin

**Affiliations:** * Vanderbilt University School of Medicine; Nashville, Tennessee, USA;; † Tennessee Department of Health, Nashville, Tennessee, USA;; ‡ Centers for Disease Control and Prevention, Atlanta, Georgia, USA

**Keywords:** Influenza, children, surveillance, epidemiologic methods, research

## Abstract

Although influenza causes more hospitalizations and deaths among American children than any other vaccine-preventable disease, deriving accurate population-based estimates of disease impact is challenging. Using 2 independent surveillance systems, we performed a capture-recapture analysis to estimate influenza-associated hospitalizations in children in Davidson County, Tennessee, during the 2003–2004 influenza season. The New Vaccine Surveillance Network (NVSN) enrolled children hospitalized with respiratory symptoms or fever and tested them for influenza. The Tennessee Emerging Infections Program (EIP) identified inpatients with positive influenza diagnostic test results through review of laboratory and infection control logs. The hospitalization rate estimated from the capture-recapture analysis in children <5 years of age was 2.4 per 1,000 (95% confidence interval 1.8–3.8). When NVSN estimates were compared with capture-recapture estimates, NVSN found 84% of community-acquired cases, EIP found 64% of cases in which an influenza rapid test was performed, and the overall sensitivity of NVSN and EIP for influenza hospitalizations was 73% and 38%, respectively.

## Abstract 2

Two surveillance systems gave a better estimate of influenza hospitalizations in children <5 years of age than either system alone.

Influenza is an important cause of acute respiratory infections and hospitalization in children (*1**–**10*). Since influenza may be indistinguishable from other respiratory and febrile illnesses, identification of infection requires diagnostic testing. Population-based studies report attack rates ranging from 15% to 42% in preschool and school children during typical outbreaks (*11**,**12*). However, defining the impact of influenza for more serious outcomes such as hospitalizations and deaths requires surveillance and testing of large populations, which may be expensive and time-consuming. Influenza surveillance systems can identify onset of disease activity, characterize viral isolates to help decide future vaccine composition, assess the impact of disease in different age and risk groups, and estimate vaccine impact (*4**–**6**,**13**,**14*). Identification of all cases of influenza or an unbiased sample of cases without regard to vaccination status is necessary to correctly measure disease impact and to assess vaccine effectiveness.

From 2003 to 2004, two independent population-based surveillance systems operated in Davidson County, Tennessee, to evaluate the impact of influenza disease in children. One prospectively tested samples from children <5 years of age who had been hospitalized with fever or respiratory symptoms. The other retrospectively identified hospitalizations for children with laboratory-confirmed influenza based on review of laboratory and infection control logs. Using data from the 2003–2004 influenza season independently generated by both systems for Davidson County residents <5 years of age, we applied a capture-recapture technique to obtain a better estimate of the total number of young children hospitalized with influenza.

## Methods

The 2 surveillance systems used in Davidson County to assess the impact of influenza disease were the New Vaccine Surveillance Network (NVSN) and the Emerging Infections Program (EIP). The Centers for Disease Control and Prevention (CDC) established the NVSN in 1999 to evaluate the incidence of acute viral respiratory infections and to assess the impact of new vaccines and vaccination policies. Influenza surveillance in the NVSN has been conducted among children <5 years of age in the inpatient setting year round since August 2000. Three sites conduct active population-based surveillance, but only the Davidson County site was included for this study. Davidson County has an estimated population of 37,813 children <5 years of age (2000 US Census). County residents <5 years of age hospitalized with respiratory symptoms or fever were enrolled 4 days per week and within 48 h of admission after informed consent was obtained. When a child was enrolled, a questionnaire was administered to parents, and 1 nasal and 1 throat swab specimen were collected from the child. These specimens were combined in a tube of veal infusion broth transport medium and delivered at ambient temperature within 1 to 2 h to the site research laboratory. Swab specimens are comparable to nasopharyngeal washes for influenza detection; however, swabs are more acceptable to families and less expensive to obtain (*15**–**18*).

Viral culture and reverse transcription–polymerase chain reaction (RT-PCR) were performed on these samples and medical charts were reviewed. To exclude nosocomial infections, NVSN excluded newborns who never left the hospital and those hospitalized in the previous 4 days. Similarly, children whose parents refused enrollment and those who were transferred from another surveillance hospital (to avoid double enrollment) were excluded. Children who were ill for >14 days did not meet our definition of acute respiratory illness, and those with fever and neutropenia were excluded because of logistic reasons. A child was considered to have influenza if the viral culture was positive or the RT-PCR result was positive on the initial test and 1 repeat test using a duplicate specimen aliquot. The results of these tests were not entered in the hospital chart and were not communicated to clinicians. NVSN performed surveillance at 3 hospitals that historically included at least 95% of all acute respiratory illness hospitalizations for children <5 years of age in Davidson County (*14*).

EIP, which was also organized and supported by CDC, was initially designed to estimate the impact of community-acquired invasive bacterial and foodborne infections through a population-based surveillance system (*19*). Because of unusual influenza activity during the 2003–2004 influenza season (*20*), EIP expanded its activities to conduct active, population-based surveillance for clinical laboratory-confirmed influenza hospitalizations in patients <18 years of age. For this analysis, only Davidson County data for children <5 years of age were included. EIP estimates the incidence of influenza hospitalizations by identifying hospitalized children with the diagnosis of influenza established by clinical laboratory testing. In Davidson County, in addition to those 3 hospitals where NVSN conducted surveillance, EIP included 7 additional hospitals that occasionally admitted Davidson County children. Hospitalized children <5 years of age with a clinical laboratory test result indicating influenza were identified and their charts were reviewed. For EIP, whether to test and which test to use were at the discretion of the attending physicians who were responsible for the child's medical care. Commercially available rapid tests, viral culture, immunofluorescence antibody staining, RT-PCR, immunohistochemical staining, and serologic analysis of paired acute-phase and convalescent-phase sera indicating a 4-fold increase in influenza antibody titer were the diagnostic techniques accepted by the EIP. A statement in the medical history that the child had a positive rapid test result for influenza performed in the outpatient setting was also acceptable. The EIP excluded children who were hospitalized >14 days after they tested positive for influenza and children whose symptom onset was >3 days after hospital admission.

A child enrolled as an influenza hospitalization by both NVSN and EIP was defined as a matched case. The identification of matched cases was determined retrospectively by comparing identified cases from the 2 systems and was based on name, date of birth, and date and place of hospitalization.

Institutional Review Boards (IRBs) of the participating hospitals and CDC approved NVSN surveillance. Since EIP influenza surveillance was considered a public health response program, it was exempt from IRB review and did not require informed consent of subjects or parents. This study was reviewed and approved by the Vanderbilt University IRB.

### Statistical Analysis

**Figure 1:**
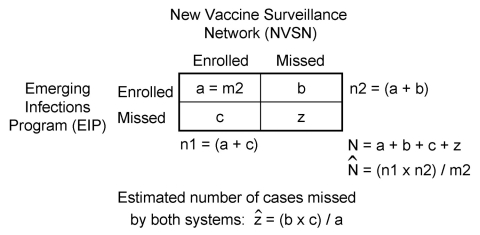
Capture-recapture estimation using data from 2 independent sources. The first surveillance system (New Vaccine Surveillance Network [NVSN]) captured n1 cases. The second system (Emerging Infections Program [EIP]) captured n2 cases, including m2 cases already captured by NVSN (matched cases). The Peterson estimator of N (total cases) is 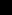
= n1 × n2/m2. The Peterson estimate implies that the estimated number of cases missed by both systems (z) = (b × c)/(a); where b is the number of enrolled cases by the EIP only, c is the number of enrolled cases by the NVSN only, and a is the number of matched cases (m2) (*21**–**25*).

We denoted as N the true total number of children <5 years of age hospitalized with influenza during the surveillance period in Davidson County. We estimated N by using the Petersen capture-recapture estimator (*21*), which we denoted 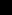
(Figure 1). The first surveillance system (NVSN) captured n1 cases from the total number of cases (N). The probability of capture is estimated by n1/N. The second system (EIP) captured n2 cases, including m2 cases that were already captured by the first system (recaptured or matched cases). The probability of being recaptured by the second system is estimated by m2/n2. When the probabilities of capture by 2 surveillance systems are independent, the probability of capture by the first system will equal the probability of recapture by the second. Equating our estimates of these probabilities and solving for N gives the Peterson estimator or 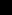 = n1 × n2/m2. This estimate assumes that the probability of being captured by 1 system does not affect the probability of being captured by the other, that the population is closed (the study population remained approximately constant and without significant migration during the study period), and that the ascertainment of influenza by the surveillance systems is valid (*21**–**25*).

Confidence intervals (CIs) for N were calculated using likelihood-ratio support intervals (*26*). The 95% CI for N consisted of all population sizes for which the log-likelihood-ratio chi-square statistic was <3.84. Since NVSN attempted to identify all cases on surveillance days, the age distribution derived from this system likely represented the true age distribution of cases. This age distribution was applied to the capture-recapture estimated total cases to derive age-specific estimates for children <6 months, 6–23 months, and 24–59 months of age.

Data collected in preparing to establish NVSN showed that admission rates for acute respiratory infections were similar for study sampling and nonsampling days. Hospitalizations per 1,000 children for NVSN were estimated by weighting the observed number of enrolled hospitalizations to account for sampling 4 days a week (Sunday 7:00 a.m. to Thursday 7:00 a.m.) and nonenrollment. This weighting factor has 2 components: sampling days by week and recruitment rate by age group and quarter of year. The first component is 7 divided by the number of days per week of enrollment, usually 4. For the second component, the quarterly enrollment rate for each of 3 age strata was calculated. The first component was divided by the second component to give the final weight, which was multiplied by the age-specific numbers of enrolled children.

Rates were calculated by dividing the weighted (NVSN) or unweighted (EIP) number of influenza hospitalizations by the population estimates for Davison County obtained from the 2000 US Census. We assumed that the population of children <6 months of age was half the number of children <1 year of age. Sensitivities of each surveillance system were calculated by dividing the rates generated by each of these systems by the rate generated through the capture-recapture estimates. Analyses were performed with Stata version 8.2 software (Stata Corporation, College Station, TX).

## Results

During the 2003–2004 nine-week influenza season in Davidson County, NVSN identified 274 eligible children admitted with acute respiratory infections or fever and enrolled 250 (91%), of whom 29 (11.6%) had influenza. Nonenrolled children included 18 whose parents were not available or refused to give consent, 3 who had non-English–speaking parents and no translator was available, 2 who were discharged before parents could be interviewed, and 1 who was missed. EIP identified 34 cases meeting its selection criteria through a systematic review of laboratory and medical records. The total number of influenza-associated hospitalizations among Davidson County residents <5 years of age detected by the 2 surveillance systems was 52, 29 for NVSN with surveillance 4 days per week and 34 for EIP with surveillance 7 days per week. Eleven children were identified in both systems (matched cases). The capture-recapture analysis estimated 38 cases missed by both systems, yielding 90 (95% CI 67–145) influenza hospitalizations of children <5 years of age. Among children identified through NVSN, 3% were admitted to an ICU compared with 6% of children identified through the EIP system.

**Figure 2:**
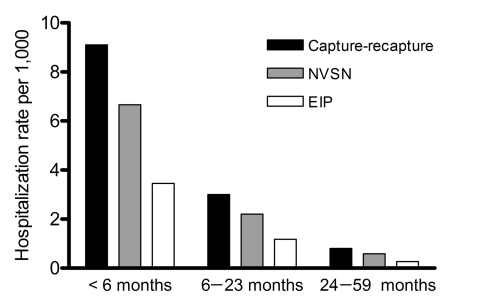
Influenza hospitalization rates in children <5 years of age by capture-recapture estimates and surveillance systems, Davidson County, Tennessee, 2003–2004 influenza season. NVSN, New Vaccine Surveillance Network; EIP, Emerging Infections Program.

The capture-recapture estimated hospitalization rate was 2.4 (95% CI 1.8–3.8) per 1,000 children <5 years of age (Table 1). Children <6 months of age had the highest hospitalization rate, 9.1 hospitalizations per 1,000 children, followed by children 6–23 months of age with 3.0 hospitalizations per 1,000 children. After weighting for sampling days and nonenrollment, the overall NVSN estimated hospitalization rate for children <5 years of age was 1.7 per 1,000, yielding an overall sensitivity of 73% compared with capture-recapture estimates (Table 2). EIP, which could only detect a clinical laboratory test with a positive result for influenza, had an estimated hospitalization rate of 0.9 per 1,000 children, yielding a sensitivity of 38% when compared with the capture-recapture estimation. (Figure 2)

**Table 1:** Estimated number of children <5 years of age hospitalized with laboratory-confirmed influenza, hospitalization rates, and rate ratios, Davidson County, Tennessee, 2003–2004 influenza season*

Age, mo	Influenza hospitalizations (95% CI)	Population	Hospitalizations per 1,000 (95% CI)	Rate ratio (95% CI))
<6	37 (27–59)	4,056	9.1 (6.7–14.5)	11.1 (6.1–20.7)
6–23	35 (27–57)	11,825	3.0 (2.3–4.8)	3.6 (1.9–6.7)
24–59	18 (13–29)	21,932	0.8 (0.6–1.3)	Referent
Total	90 (67–145)	37,813	2.4 (1.8–3.8)	

*Capture-recapture estimation. Age distribution derived from the New Vaccine Surveillance Network. CI, confidence interval.

**Table 2:** Influenza hospitalization rates per 1,000 children <5 years of age and sensitivity of system compared to capture-recapture estimates, Davidson County, Tennessee, 2003–2004 influenza season*

Age, mo	Hospitalization rates (%) per 1,000 children		Sensitivity (%) compared to capture-recapture estimates	
	NVSN†	EIP	NVSN	EIP
<6	6.66	3.45	72.97	37.84
6–23	2.20	1.18	74.29	40.00
24–59	0.59	0.27	72.22	33.33
Total	1.75	0.90	73.33	37.78

*NVSN, New Vaccine Surveillance Network; EIP, Emerging Infections Program. †Estimation corrected for nonsurveillance days and nonenrolled children.

Both surveillance systems sought to estimate the total number of influenza hospitalizations in county residents <5 years of age, but selection criteria differed. Children that were missed by 1 system and detected by the other were identified (Table 3). For NVSN, 16 (70%) of 23 missed case-patients were identified during nonsurveillance days and therefore not enrolled. In addition, 1 child's parent refused enrollment. By design, NVSN rates were adjusted for missed days of surveillance and nonenrolled cases (*14*). However, 6 patients hospitalized on surveillance days were not included in the NVSN rate estimation. Three of these 6 children had been hospitalized in the previous 4 days and were not enrolled because they met NVSN exclusion criteria. On admission, 1 child was enrolled and tested negative for influenza by viral culture and PCR. Exclusion of these 4 cases from the capture-recapture analyses resulted in an estimated rate of 2.1 per 1,000 community-acquired influenza hospitalizations, and an NVSN sensitivity of 84%. Two additional children, 1 who had diarrhea and thus did not meet inclusion criteria, and another who met selection criteria but was missed, were not enrolled.

**Table 3:** Nonenrolled influenza patients by surveillance system, Davidson County, Tennessee, 2003–2004 influenza season*

Case-patients not enrolled by NVSN	No.
Hospitalized on nonsurveillance days	16
Refused enrollment	1
Hospitalized in past 4 days, excluded	3
Enrolled, tested negative for influenza at admission	1
Hospitalized with nonrespiratory symptoms, excluded	1
Missed	1
Total	23
Case-patients not enrolled by EIP	
No influenza tests ordered	12
Rapid test for influenza done, negative result	5
Missed	1
Total	18

*NVSN, New Vaccine Surveillance Network; EIP, Emerging Infections Program.

For EIP, 12 (67%) of 18 patients identified only through NVSN were not enrolled because no influenza test had been ordered by their physician. In addition, 5 patients were tested with influenza rapid tests but negative results were obtained. Only 1 child whose chart indicated a positive influenza rapid test result was not identified by EIP surveillance. We repeated the capture-recapture analysis that included only children who had a clinical laboratory test for influenza. This analysis resulted in an influenza hospitalization rate of 1.4 per 1,000 children <5 years of age. The sensitivity of the EIP for detecting influenza was 64% among children who had a rapid test performed.

Influenza viral culture and RT-PCR were performed on cultures from all children enrolled by NVSN. The diagnosis was made by culture alone in 7%, RT-PCR alone in 21%, and by both in 72%. All patients detected by EIP had a positive result in a commercially available rapid test. The most common test (59%) was Directigen Flu A + B (Becton Dickinson Diagnostic Systems, Sparks, MD, USA), a membrane-based enzyme immunoassay.

## Discussion

With fluctuating vaccine supplies, variable onset and severity of influenza seasons each year, and new recommendations for use of influenza vaccine in children, an accurate, informative influenza surveillance system is greatly needed. During the 2003–2004 influenza season, analysis of data from 2 independent surveillance systems, both of which included children <5 years of age, provided better estimates of hospitalization rates since it accounted for those cases undetected by each system.

NVSN attempted to enroll all potential influenza admissions on surveillance days and used the most sensitive and specific diagnostic tests to detect influenza (*18*). Reliance on viral culture alone for influenza diagnosis would have missed 21% of NVSN cases, whereas use of RT-PCR would have missed only 7%. The combination of these techniques increased the detection of influenza by the NVSN. In addition, nonsurveillance days and children whose parents refused enrollment were taken into account in NVSN rate calculations. With intense surveillance, NVSN detected 73% of influenza hospitalizations estimated by the capture-recapture analysis. Exclusion of the 4 possible nosocomial cases increased the sensitivity of NVSN to 84%. NVSN selection criteria were established to specifically exclude nosocomial cases, including children discharged within 4 days of readmission. One child was enrolled by NVSN and tested negative for influenza virus on admission but had a clinical laboratory test result indicating influenza after >1 week of hospitalization. Three other children were excluded by NVSN criteria because of a recent hospitalization. However, with available information, whether these were nosocomial infections could not be determined. Based on results of the capture-recapture analysis, NVSN modified its methodology in subsequent years to include children recently hospitalized.

The EIP surveillance system sought to find all hospitalized children with positive clinical laboratory test results for influenza. One limitation of the EIP was that influenza ascertainment relied on a diagnostic test ordered by the physician. Another limitation was the sensitivity of the rapid influenza detection tests. When tests were not ordered or yielded false-negative results, influenza cases were undetected. EIP surveillance was cheaper and logistically simpler to implement than NSVN. Since EIP was considered a public health response program in Tennessee, it did not require parental informed consent. However, EIP will underestimate the impact unless combined with additional information on the proportion of patients with true cases who are tested and the sensitivity of the diagnostic tests used. During its first year of influenza surveillance in Davidson County, EIP missed only 1 patient who could have potentially been detected. However, because the rapid influenza antigen test, the only clinical laboratory influenza test used in these patients, was less sensitive than RT-PCR plus viral culture, the estimated sensitivity of EIP for children who actually had clinical laboratory tests performed was 64%. When compared with viral culture, these tests have a sensitivity ranging from 44% to 95% and a specificity ranging from 76% to 100% (*27**–**30*). Although the ability of EIP to detect influenza cases was dependent on these test characteristics, the primary reason for EIP's underestimation of rates was that diagnostic tests for influenza were not ordered for most children admitted with influenza. The capture-recapture analysis indicated that only 38% of children <5 years of age hospitalized with influenza were correctly identified by routine diagnostic tests. Thus, not detecting influenza during hospitalization resulted not only in underestimating the impact of influenza, but also in providing limited opportunity for appropriate antiviral therapy.

Capture-recapture methods emerged as an adaptation of techniques used by wildlife researchers to obtain better counts of difficult-to-enumerate wild animals. The simplest technique uses 2 samples or lists. Using the number of individuals caught in each sample (captures) and the number of subjects from the first sample that were captured again by the second sample (recaptures), one can estimate the number of subjects not caught in either sample, thus providing an estimate of the total population size (*31**–**34*). The estimation directly accounts for different capture probabilities of each sample, and allows one to obtain estimates using 1 source that operated 4 days a week (NVSN) and the other that operated continuously (EIP).

Since being identified in 1 system did not influence the possibility of identification in the other system, the independence of the 2 systems was assumed. The independence assumption could have been violated if some factor, such as severity of influenza illness or viral load, varied among subjects and the likelihood of detection increased in both systems with increasing severity or viral load. In this case, the Peterson method would underestimate the true population size. In addition, both systems would likely miss children with very low or no influenza viral loads, such as those admitted late in the course of illness. This would also underestimate the true rates.

No significant migration occurred in Davidson County during the study, and the study population was restricted to county residents and assumed to be closed. This study was conducted during a single influenza season and there were relatively small numbers of cases identified, which precluded detailed subgroup analyses. However, the final estimation of influenza hospitalization rates was consistent with previous reports of the 2003–2004 influenza season and with previous research indicating that children <24 months of age have hospitalization rates similar to those of persons >65 years of age (*11**,**12**,**20**,**35*). This estimation also highlights the great impact of influenza, particularly in children <6 months of age during a moderately severe influenza season. Current vaccines are poorly immunogenic in this age group and have not been approved for these children. Thus, vaccination of household contacts and out-of-home caregivers of children <6 months of age is recommended. Additional influenza vaccination of children 6–23 months of age has also been recommended to limit their exposure (*35**,**36*). As immunization rates in families of young children increase and routine vaccination for children 6–23 months of age is implemented, surveillance systems must be in place to effectively measure the impact of these preventive strategies.

Combined NVSN and EIP systems analyzed with the capture-recapture approach appear well suited to this important task. EIP is a simpler and cheaper system for identifying children with influenza. Although EIP could estimate rates more accurately by adjusting for known sensitivities and specificities of clinical diagnostic tests, without information on the frequency of diagnostic testing, it would be impossible to determine and adjust for the proportion of influenza this system captures. Thus, the degree of underascertainment would be unknown. In addition, such diagnostic testing will likely change over time, making year-to-year comparisons of disease impact difficult. NVSN attempted to estimate the true impact of influenza hospitalizations by testing all children with specific admission criteria, adjusting for nonenrollment and nonsurveillance days, and providing an unbiased sample of influenza-positive children for further analyses such as vaccine effectiveness estimates. However, this system also underestimated the total influenza impact. The combined systems gave the best estimate of disease impact.

Currently, no population-based surveillance systems are available to monitor the influenza vaccine program in adults. Using a combination of 2 systems similar to NVSN and EIP could be a model for surveillance of influenza in adults. The more expensive and labor-intensive NVSN-type surveillance could be conducted at representative hospitals in a geographic area for limited periods during the influenza season (e.g., 1 day/week at each hospital). The EIP-type surveillance system could attempt to identify all persons admitted with influenza identified through routine testing. Capture-recapture methods could be used to more accurately estimate serious influenza impact. Comparison of patients could determine whether those identified through cheaper EIP methods were representative of all patients with respect to important characteristics such as influenza vaccination status and severity of disease. Capture-recapture techniques should be considered as methods to best use limited resources for essential surveillance activities.
